# L-Carnitine ameliorates the liver by regulating alpha-SMA, iNOS, HSP90, HIF-1alpha, and RIP1 expressions of CCL4-toxic rats

**DOI:** 10.22038/IJBMS.2020.47711.10990

**Published:** 2021-02

**Authors:** Derya Karabulut, Ali Tugrul Akin, Murat Unsal, Ayça Lekesizcan, Tuğçe Merve Ozyazgan, Didem Barlak Keti, Birkan Yakan, Görkem Ekebas

**Affiliations:** 1 Department of Histology-Embryology, Faculty of Medicine, Erciyes University, Kayseri, Turkey; 2 Department of Biology, Faculty of Science, Erciyes University, Kayseri, Turkey; 3 Department of Biochemistry, Faculty of Medicine, Erciyes University, Kayseri, Turkey; 4 Department of Pathology, Faculty of Veterinary, Erciyes University, Kayseri, Turkey

**Keywords:** Alpha-SMA, CCL4, HIF-1alpha, HSP90, Inos, RIP1

## Abstract

**Objective(s):**

Carbon tetrachloride (CCL_4_) toxicity triggers fibrosis, activating various mechanisms within the cell. We aimed to create damage with CCL_4_ and investigate the effectiveness of L-carnitine on the mechanisms we identified.

**Materials and Methods:**

Forty rats were divided into 5 groups with equal number of rats in each group. Group I: Control group, Group II: L-carnitine group, 200 mg/kg L-carnitine twice a week, Group III: CCL_4_ group, 0.2 ml/100 gr CCL_4_, IP, dissolved in olive oil 2 times a week during 6 weeks; Group IV: L-carnitine + CCL_4_ group, 200 mg/kg L-carnitine 24 hr before 0.2 ml/100 g CCL_4_ application twice a week; Group V: CCL_4_ + L-carnitine, 200 mg/kg L-carnitine half an hour after 0.2 ml/100 g CCL_4_ application. The liver was evaluated histologically. Immunohistochemically stained with α-SMA, iNOS, HSP90, HIF-1α, and RIP1. TNF-α, TGF-β, AST, ALT, ALP, and GGT measurements were evaluated.

**Results:**

In the classical lobule periphery, an increase in lipid accumulation and a decrease in glycogen accumulation were observed. After immunohistochemical measurements and biochemical analyzes, an increase in the expression density of all proteins was observed in group III. In group IV and V, an improvement in tissue and a decrease in protein expression densities were observed.

**Conclusion:**

iNOS serves as a free radical scavenger in response to damage caused by increased toxicity of α-SMA, HSP90, and HIF-1α. Especially, increased RIP1 level in the tissue indicates the presence of necrosis in the tissue after CCL_4_-toxicity. Supplementing the amount of endogenous L-carnitine with supplementation provides a significant improvement in the tissue.

## Introduction

Fibrosis is a basic connective tissue lesion formed as a result of increased fibrillar extracellular matrix in the organ or tissue. This is a chronic inflammatory process that often occurs in pathological conditions (vascular, metabolic, and tumor pathology) (1). Fibrosis occurs as a result of the balance between the extracellular matrix production (fibrogenesis) and its destruction (fibrolysis) (2). Liver fibrosis is the most common chronic liver damage and is an irreversible process, especially after it turns into cirrhosis (3). Various diseases, including alcohol, drugs, viruses, and genetic disorders, can cause this and are especially caused by chronic liver damage (4). Hepatocellular damage can be directly caused by toxicity in the liver. Chloroform, various antineoplastic antibiotics, tannic acid, and tetracyclines are included in this group. In studies, carbon tetrachloride (CCl_4_) or etionin is applied to the animals in order to create primary hepatotoxicity. CCl_4_ is applied to experimental animals at different doses and times to create liver fibrosis in various studies (5-8). Hepatic stellate cells (HSCs) multiply, migrate, and become visible to contractile myofibroblasts in the fibrogenic liver (9). The most used marker of myofibroblast is α-smooth muscle actin (α-SMA) (10). 

Nitric oxide (NO) is a free radical that is synthesized from L-arginine through action of nitric oxide synthase enzymes (NOS) and can be covalently bonded with other molecules due to its unpaired electron in its final orbit (11, 12). Inducible nitric oxide synthase (iNOS) isoform is induced in a wide range of tissues and cell types to which cytokines and bacterial products are exposed (12). Heat shock proteins (HSP) are a family of proteins that are expressed at low levels under normal conditions but are induced against cellular stress in situations such as heat shock, hypoxia, toxic agents, and food starvation. In hepatocellular carcinoma, apoptosis plays an important role in therapeutic resistance, invasion, and metastasis (13). HSP90 is one of the most abundant proteins in cells. In recent years, it has been shown that receptor-interacting protein (RIP)-mediated necrosis is associated with HSP90 (14). Impaired hepatic blood flow and sinusoidal fibrin accumulation in fibrosis cause the development of hepatocellular hypoxia by affecting portal-systemic vessels. Hepatocellular hypoxia stimulates the activation of hypoxia-inducible factor-1α (HIF-1α) (15). It is important to evaluate the relationship between the above-mentioned markers and necrosis after CCl_4 _toxicity. Therefore, this study is an innovative perspective.

L-carnitine is a white, water-soluble substance with good thermostability. In mitochondria, it fulfills its catalytic function in the formation of fatty acids and its metabolic function by acting as a buffer for excessive acyl residue (16). It also protects the cell membrane and DNA against damage from free oxygen radicals, while preventing protein oxidation and lactate oxidative damage (17). Therefore, the potential power of L-carnitine is still used in various studies. 

In this study, we aimed to evaluate the effects of L-carnitine in liver damage caused by CCl_4_. Hence, we evaluated various mechanisms that may occur as a result of tissue damage. We especially showed the effect of L-carnitine on necrosis, and we investigated its relationship with other mechanisms. We aimed to evaluate the protective and therapeutic effects of L-carnitine against these mechanisms and damage caused by CCl_4_ by histological, biochemical, and immunohistochemical methods.

## Materials and Methods

***Animals***

In this study, 40 adult Spraque Dawley male rats (obtained from Hakan Çetinsaya Experimental and Clinic Research Center, Erciyes University, Kayseri, Turkey) were used. Rats were divided into groups and placed in well ventilated plastic cages. Standard procedure was applied without restricting access to feed and water needs. Rats were kept in a room with a 12 hr light:dark cycle. All animals received humane care according to standard rules, and all ethical rules have been followed. Ethical approval for the study was obtained from Erciyes University Animal Research Local Ethics Committee (date: 14/02/2018, decision no: 18/034). 

***Experimental design***

The rats were randomly assigned to five groups of eight . Group I: served as the Control group (n=8); Group II: L-carnitine group (n=8), 200 mg / kg L-carnitine twice a week (18); Group III: CCl_4 _group (n=8), 0.2 ml/100 g CCl_4_, IP, dissolved in olive oil 2 times a week during 6 weeks (19); Group IV: L-carnitine + CCl_4_ group (n=8), 200 mg / kg L-carnitine 24 hr before 0.2 ml/100 g CCl_4 _application twice a week; Group V: CCl_4_ + L-carnitine group (n=8), 200 mg/kg L-carnitine half an hour after 0.2 ml/100 g CCl_4_ application.

***Chemicals***

Carbon tetrachloride (Sigma-Aldrich, St. Gallen, Switzerland) was used intraperitoneally as an inducer of liver damage. L-carnitine (Sigma-Tau, Pomezia, Italy) was used as a protective and therapeutic substance in the experiment.

***Histological examination***

At the end of the experiment, rats were anesthetized using anesthetic agents (ketamine (75 mg/kg) + xylazine (10 mg/kg)). Liver tissues were taken in 4% formaldehyde solution. Then the routine light microscopic procedure was applied. For this procedure, dehydration was first applied to the tissues. Then it was made transparent by holding in xylene and fixed blocks were made with paraffin. Sections were taken from paraffin blocks and Hematoxylin-Eosin and stained with periodic acid-Schiff . Sections were examined under a light microscope (Olympus BX51, Center Valley, PA, USA) (20).

***Hematoxylin-eosin staining***

Liver tissue sections were incubated at 58 °C for 2 hr. First, they were deparaffinized by xylene. Then they were rehydrated and made suitable for painting. Sections washed with water were first kept in hematoxylin. They were then washed again and treated with eosin. Sections were closed again by passing through alcohol and xylene.

***Periodic acid schiff staining***

Paraffin sections were incubated at 58 °C for 2 hr. After deparaffinization and rehydration, they were washed with water. Periodic acid was kept for 10-15 min, after washing with water, they were placed in the Schiff solution for 20 min at room temperature in the dark. After washing under running water, they were kept in the Hematoxylin solution. Acid-alcohol was applied and washed. After dehydration and clearing they were examined under the microscope.

***Immunohistochemistry***

Expressions of α-SMA (Anti α-SMA antibody, Bioss, USA), iNOS (Anti NOS-2/iNOS antibody, Bioss, USA), heat shock protein 90 (HSP90) (Anti HSP90 alpha antibody, Bioss, USA), hypoxia-inducible factor 1 alpha (HIF-1α) (Anti HIF-1α Antibody, Bioss, USA) and receptor-interacting protein kinase 1 (RIP1) (Anti-RIP1 antibody, Bioss, USA) in liver tissue were determined. The procedure was performed under identical conditions for all sections. Deparaffinization and dehydration processes were performed on paraffin sections, respectively. After washing these sections with water, antigen retrieval (with citrate buffer) was applied. Slides were left at room temperature for 20 min. Phosphate-buffered saline (PBS) was used as a washing solution. Sections were inhibited by 3% H_2_O_2 _in methanol for 10 min. The staining kit (Lab Vision, Ultra Vision Detection System Large Volume, Anti-Polyvalent Thermo Scientific HRP) was used for the next stages according to manufacturer’s instructions. 3,3P-diaminobenzidine tetrahydrochloride (DAB) was applied to the slides and counterstained with hematoxylin. Under a light microscope (Olympus BX51, Center Valley, PA, USA) the images were obtained. Ten different areas were evaluated from each of the subjects in terms of the positive cells using the Image J program (21). 

***Biochemical analysis***

Liver samples were taken into empty tubes to obtain serum. They were then homogenized and centrifuged at 1.509 g for 10 min. The resulting serum was used for determination of transforming growth factor-beta 1(TGF- β) and tumor necrosis factor-alpha (TNF-α) (ELISA kits respectively, E-EL-R0084 and E-EL-R0019, 96 Wells kit, Elabscience Biotechnology Co., Ltd.) using ELISA kits. Elabscience Biotech rat ELISA kit protocols were used. Alanine aminotransferase (ALT), aspartate aminotransferase, alkaline phosphatase (ALP), and gamma-glutamyl transferase (GGT) values of blood serum samples taken at the end of the experiment were analyzed by the service in Erciyes University Central Biochemistry Laboratory (20).

***Statistical analysis***

The study data were obtained using GraphPad (Prism 7.00 for Mac, GraphPad Software, La Jolla, California, USA). It was first determined whether these data were normally distributed or not. D’Agostino Pearson omnibus test was used for this purpose. In the case of the normal distribution, quantitative variables were compared using one-way analysis of variance and Tukey’s *post hoc* test. Kruskal-Wallis test and Tukey’s *post hoc* test were used for comparing the quantitative with the abnormal distribution. The data were expressed as the mean of normalized data±standard deviation of the mean. *P*<0.05 was considered as statistically significant.

## Results

***Histological results***

**Figure 1:**
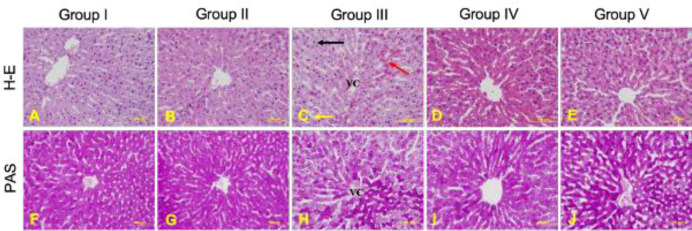
Liver tissues of all experimental groups. H-E staining of liver tissues (A-E). (A) Liver of group I (control group), (B) Group II (L-carnitine group), (C) Group III (CCL_4_ group) (D) Group IV (L-carnitine + CCL_4_ group), (E) Group V (CCL_4_ + L-carnitine group). Sinusoidal dilation (yellow arrow), lipid droplets (black arrow), eosinophilic hepatocytes (red arrow). PAS staining of liver tissues (F-J). (F) Liver of group I (control group), (G) Group II (L-carnitine group), (H) Group III (CCL_4_ group), (I) Group IV (L-carnitine + CCL_4_ group), (J) Group V (CCL_4_ + L-carnitine group). Scale bar 100 µm

H-E: Hematoxylin-Eosin; VC: Vena centralis; CCL_4_: Carbontetrachloride; PAS; Periodic Acid Schiff

Liver tissue had a normal structure in group I. Group II liver tissue structure was similar in appearance to group I. PAS staining in hepatocytes was intense in both group I and group II. In group III disorganized cells were observed extending from the vena centralis and dilation of the sinusoids. In addition, lipid droplets were observed in hepatocytes at the periphery of the classical lobule in this group. Also, numerous eosinophilic hepatocytes were observed. In Group III, a decrease in the PAS staining intensity was observed. This reduction was mostly in the periphery of the classical lobule. It was determined that there were improvements in tissue structure in liver sections belonging to groups IV and V. In both groups, the cell cords were more regular and sinusoids were more normal. There was also a decrease in the amount of lipid droplets in the classical lobule periphery. PAS staining was more intense in both group IV and group V compared with group III. H-E and PAS staining of all groups are given in Figure 1. 

***Immunohistochemistry results***

**Figure 2:**
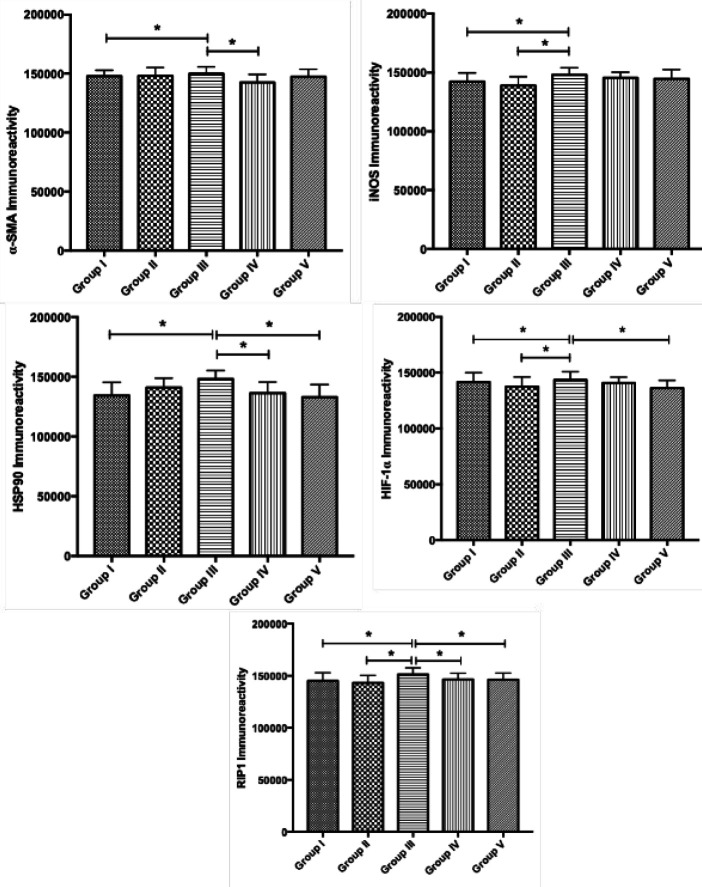
α-SMA, iNOS, HSP90, HIF-1α, and RIP1 immunoreactivity results. Values are presented as means±SD. * *P*<0.001 compared with both Group I and Group III

**Figure 3:**
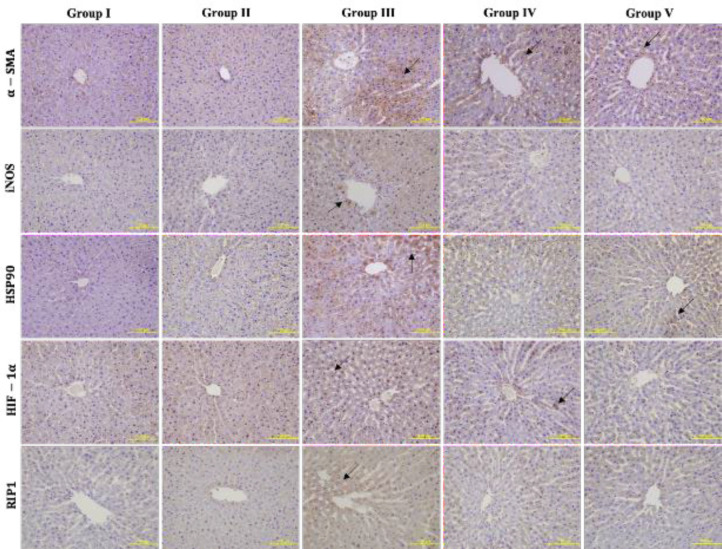
α-SMA, iNOS, HSP90, HIF-1α, and RIP1 immunohistochemical staining. The expression intensities of the proteins in the groups are indicated by arrows. Scale bar 100 µm

α-SMA, iNOS, HSP90, HIF-1α, and RIP1 immunoreactivities were determined in the liver tissue (Figure 2). In group III, the expressions of all proteins in liver sections increased compared with group I. α-SMA expression was significantly decreased in group IV compared with group III. Also, α-SMA expression was more intense in the classical lobule periphery. iNOS expression increased statistically in group III compared with group I. iNOS expression decreased in groups IV and V, but this decrease was not significant. HSP90 expression increased in group III compared with group I. Its expression decreased significantly in group IV and V compared with group III. HIF-1α expression increased significantly in group III compared with both groups I and II. There was a statistically significant decrease between groups IV and V with group III. This decrease was significant in group V. RIP1 expression increased significantly in group III compared with groups I and II. This increase decreased significantly in Groups IV and V. The expression intensity pictures are given in Figure 3.

***Biochemical results***

TNF-α and TGF-β levels in the liver were determined at the end of the experiment. TNF-α levels were significantly increased in both group III and group IV compared with group I. Group V was similar to group I TNF-α level. TGF-β levels differences were not statistically significant among all groups. AST, ALP, and GGT levels increased in group III compared with control, but this increase was not statistically significant (*P*<0.05). ALT level decreased in group III; compared with group I, this decrease was not statistically significant. AST, ALT, ALP, and GGT levels did not differ statistically in groups IV and V. Results of biochemical analysis of all groups were compared with each other, and the data obtained are shown in Table 1.

## Discussion

More than six months of dysfunction in the liver, which performs metabolic activities such as bile excretion, detoxification of harmful products, synthesis of various proteins, causes the appearance of chronic liver diseases (22). Therefore, it is important to identify ways of diagnosis and treatment in the early period. Different mechanisms are triggered by using various proteins, markers, and enzymes in the cell during disease formation. If no precautions are taken, this primarily recyclable process usually continues irreversibly. In this study, we aimed to create a reversible damage process in the liver by using CCl_4_ and to investigate the effect of L-carnitine against this damage. According to our results, L-carnitine provides a significant improvement in the liver as a protective and therapeutic agent against CCl_4 _toxicity. Hepatocytes, sinusoidal spaces, infiltration, glycogen storage disorders are observed in the liver for various toxic reasons (20, 23). L-Carnitine showed both protective and healing effects against this liver damage. These effects are realized under the influence of various mechanisms.

It has been reported that the level of TNF-α increases after non-lethal dose CCl_4_ administration and this increase is associated with the release of increased hepatic enzymes from hepatic damage (24). In our study, increased TNF-α was observed in group III compared with group I. Collagen up-regulation and HSCs (myofibroblasts) activation after CCl_4_ are known, so α-SMA is directly related to fibrosis (25). Collagen production as a result of the differentiation of myofibroblasts after the onset of fibrosis is evidence of TGF-β stimulation. Collagen production is an irreversible process and continues even if TGF-β is removed from the environment (26). In our study, a statistically significant increase in α-SMA expression compared with group I can demonstrate evidence of a fibrotic process in tissue. TGF-β increase is also parallel to this situation. We observed an increase in AST, ALP, and GGT values. These enzymes have been shown to increase as a result of the loss of endogenous antioxidant capacity and hepatocyte membrane integrity in the tissue following CCl_4_ application (27). In our study, the increase of enzyme and TNF-α, TGF-β and α-SMA increases are compatible with each other. The liver damage model created with CCL_4_ is associated with oxidative stress and lipid peroxidation. NO serves as both a prooxidant and a free radical scavenger (28). iNOS is responsible for the overproduction of NO, this increase causes increased toxic damage to liver cells (29). We found an increase in iNOS expression in group III. Besides, HSP90 has been reported to be necessary for liver damage caused by CCl_4_ toxicity (30). HSP90 expression increased in group III. HSP90 is the most abundant member of HSPs required for cell development, cycle, and other biological processes (14). Expressions of both iNOS and HSP90 may have increased against CCl_4_ toxicity in group III. Considering the necessity of iNOS as a free radical scavenger and HSP90 against CCl_4_-toxicity, we think that these increase as a precaution by cells against fibrosis, which is beginning to develop. Because the development of fibrosis in the tissue is associated with inflammation and angiogenesis, and HIF-1α plays an important role. There was a strong correlation between tissue angiogenesis with HIF-1α expression (31). These results make us think that iNOS and HSP90 may play a restorative role in order to eliminate the increased expression of α-SMA and HIF-1α in group III. HSP90 inhibition is known to promote apoptosis, which some studies have also shown to be associated with necroptosis (32). It is regulated by necrosis or necroptosis receptor-interacting protein 3 (RIP3). Necroptosis induces receptor-interacting protein1 (RIP1) and RIP3 expressions (14). RIP1 connects to and activates RIP3 by generating signals that cause necrotic cell death (33). When the expression of HSP90 decreases, RIP1 and TNF-α expression has also been shown to decrease (14). Our study demonstrated that RIPK1 expression was significantly elevated in group III. This result is parallel to the HSP90 increase. Therefore, when HSP90 increases, RIP1 expression may also be increased.

L-carnitine is an amino acid that is synthesized by methionine and lysine in the liver, carrying fat to mitochondria and contributing to energy production (5). It has been frequently used in liver fibrosis studies due to its synthesis in the liver and its role in the transport of fatty acids (34-36). We have shown that L-carnitine has both protective and therapeutic effects on AST, ALT, ALP, and GGT enzymes. Also, it was concluded that TGF-β and TNF-α levels were also effective in both group IV and group V compared with group III. Biochemical analysis showed decreases and increases among groups, but these were not significant in general. This may be due to the number of rats in the groups (n = 8). In these groups compared with group III, α-SMA, iNOS, HSP90, HIF-1α, and RIP1 expressions decreased. However, statistically significant decreases in group IV were in α-SMA, HSP90, and HIF-1α expressions. Likewise, the significant statistical decreases in group V were in HSP90, HIF-1α, and RIP1 expressions. We believe that the improvements in expression density in these groups treated with L-carnitine were improved in insufficient endogenous L-carnitine levels in the presence of exogenous L-carnitine. Because these proteins are components of various mechanisms that occur under stress conditions. Therefore, due to the reduced stress in the presence of L-carnitine, improvements in the expression of these proteins may have been observed. Carnitine is found in the organism in free carnitine or acylcarnitines forms. Balanced carnitine homeostasis is provided by carnitine-dependent enzymes and plasma membrane transporters. The main function of L-carnitine is the transport of fatty acids from the cytosol into the mitochondria matrix for beta-oxidation and ATP production (37). Change in endogenous L-carnitine levels due to any toxicity in liver cells triggers liver degeneration. Disruption of fatty acid metabolism causes changes in the expression of molecules and compounds that play a role in various mechanisms in the cell. Therefore, exogenous L-carnitine against toxic agents such as CCl_4_ can be important in regulating cell function and maintaining carnitine homeostasis. We found that L-carnitine in group IV and V reduced increased HIF-1α expression against CCl_4 _toxicity. HIF-1α regulates the production of growth factors in other cell types during the liver degeneration process. For this reason, HIF-1α is activated in hypoxic hepatocytes and causes an increase in various profibrotic mediators (38). After CCl_4_-toxicity, L-carnitine has been reported to have a restorative effect on both histopathological and liver enzymes, as well as reduce α-SMA (34). In parallel with the decrease of HIF-1α expressions in group IV and V, α-SMA, HSP90, and RIP1 expressions decreased significantly. This can be shown as a marker that L-carnitine reduces the fibrogenic process that begins in the tissue. iNOS immunoreactivity decreased in groups IV and V. L-carnitine is reported to prevent oxidative stress by regulating NO production (39). Increased iNOS in group III as a free radical scavenger may have decreased the level of iNOS due to L-carnitine decreasing the fibrotic process. But this was still not statistically significant. This may be related to the L-carnitine dose.

α-SMA: α-Smooth muscle actin; iNOS: Inducible nitric oxide synthase; HSP90: Heat shock protein-90; HIF-1α: Hypoxia-inducible factor; RIP1: Receptor-interacting protein 1

α-SMA: α-Smooth muscle actin; iNOS: Inducible nitric oxide synthase; HSP90: Heat shock protein-90; HIF-1α: Hypoxia-inducible factor; RIP1: Receptor-interacting protein 1

**Table 1:** ELISA results of TNF-α and TGF-β levels in all groups. Serum AST, ALT, ALP, and GGT levels of all groups

Groups	Group I	Group II	Group III	Group IV	Group V
TNF-α	53,01±23,46	53,21±26,05	102,7±19,95**	95,92±32,53*	65,26±29,48
TGF-β	25,85±16,66	28,54±13,56	31,12±13,69	22,73±7,97	30,89±14,6
AST	102,3±9,067	98,57±10,22	110,5±12,65	108,9±14,69	107,6±18,7
ALT	62,38±8,03	60,75±5,11,71	55,5±4,08	61,75±8,95	52,57±8,84
ALP	188,7±15,59	204,7±16,28	201,3±18,48	189,7±22,07	174,5±23,13
GGT	1,71±1,49	1,57±0,78	2,00±2,23	1,75±0,70	2,00±1,15

All groups were compared with each other. Values are given as mean±standard deviation. *P*<0.05 was considered significant

*P<0.0288. ** *P*<0.0097

TNF-α: Tumor necrosis factor-alpha; TGF-β: Transforming growth factor-beta; AST: Aspartate aminotransferase; ALT: Alanine aminotransferase; ALP: Alkaline phosphatase; GGT: Gamma-glutamyl transferase

## Conclusion

As a result, an increase in the expression of various molecules was observed after CCl_4_-toxicity in the tissue. It is noteworthy that HSP90, which is known to induce apoptosis, stimulates necrosis due to the increase of RIP1. We think it accompanies this in HIF-1α, but iNOS serves as a free radical scavenger. L-carnitine inevitably regresses by acting on the lipid accumulation and fibrotic process that started after CCl_4_ toxicity, thanks to its properties. As a protective and therapeutic, it corrected the tissue biochemically and histologically. The use of L-carnitine as a supplement in diseases such as enzyme deficiency and fatty liver shows that it is a product that can prevent the liver from entering the irreversible process.
